# The Exergy Losses Analysis in Adiabatic Combustion Systems including the Exhaust Gas Exergy

**DOI:** 10.3390/e24040564

**Published:** 2022-04-18

**Authors:** Senda Agrebi, Louis Dreßler, Kaushal Nishad

**Affiliations:** 1 Institute of Reactive Flows and Diagnostics, Technical University of Darmstadt, 64287 Darmstadt, Germany; dressler@ekt.tu-darmstadt.de (L.D.); nishad@ekt.tu-darmstadt.de (K.N.); 2 Institute of Energy and Power Plant Technology, Technical University of Darmstadt, 64287 Darmstadt, Germany; 3 Mechanics, Modelling Energy and Materials Unit (M2EM), National School of Engineers of Gabes, Gabes 6029, Tunisia

**Keywords:** Eulerian stochastic field method, FGM, entropy generation analysis, exhaust gases exergy, flames E and F

## Abstract

The entropy generation analysis of adiabatic combustion systems was performed to quantify the exergy losses which are mainly the exergy destroyed during combustion inside the chamber and in the exhaust gases. The purpose of the present work was therefore: (a) to extend the exergy destruction analysis by including the exhaust gas exergy while applying the hybrid filtered Eulerian stochastic field (ESF) method coupled with the FGM chemistry tabulation strategy; (b) to introduce a novel method for evaluating the exergy content of exhaust gases; and (c) to highlight a link between exhaust gas exergy and combustion emissions. In this work, the adiabatic Sandia flames E and F were chosen as application combustion systems. First, the numerical results of the flow and scalar fields were validated by comparison with the experimental data. The under-utilization of eight stochastic fields (SFs), the flow field results and the associated scalar fields for the flame E show excellent agreement contrary to flame F. Then, the different exergy losses were calculated and analyzed. The heat transfer and chemical reaction are the main factors responsible for the exergy destruction during combustion. The chemical exergy of the exhaust gases shows a strong relation between the exergy losses and combustion emission as well as the gas exhaust temperature.

## 1. Introduction

Concerns over improving the energy efficiency of combustion systems have been increasing in recent decades due to limited fossil fuel resources combined with the urgent need for reducing pollutant emissions. To optimize energy efficiency, it is essential to minimize the thermodynamic irreversibilities associated with transport phenomena in the system. In this context, the second law of thermodynamics provides a strong foundation to quantify those irreversibilities responsible for the destruction of exergy, also called the net rate of energy degradation and thus, a reduction in energy efficiency. Several studies were carried out for more exergy-efficient combustion [1,2,3,4,5]. Exergy losses in combustion systems consist of three main parts: the exergy destruction during combustion; the exergy transfer through heat transfer to the ambient; and the exergy of exhaust gases. The evaluation of these losses in the vast majority of the literature was essentially conducted experimentally and for internal combustion engines [6,7,8,9,10,11,12,13,14,15,16,17,18]. It was found that irreversibilities mainly originate from the combustion process with the engine operation [6,7,8,9,10,11,12,13,14,15,16,17,18] and it ranges from 50% to 60% of the total exergy input [19]. The exergy content of the exhaust gases ranges from 10% to 20% of the total input exergy [19,20], and the exergy lost through heat convection from the control volume to the ambient does not exceed 10% [19].

The exergy destroyed during combustion, representing the major part of exergy losses, is related to the irreversibilities associated with the different processes involved in combustion such as the heat transport, mechanical dissipation, mass and species diffusion, chemical reactions, phase change, and inelastic material deformation [1,2,3,4,5]. Such irreversibilities vitiate the available work into internal energy in the system, leading to an increase in system entropy [1,2,3,4]. It is worth noting that this increase in entropy causes the deterioration of the thermodynamic performance of the system. Thus, the estimation of the exergy inside combustion is performed through entropy generation. In turbulent reacting flows, such a phenomenon is highly unsteady and requires unsteady numerical simulation methods for reliable description. Safari et al. [21,22] were the first to employ Large Eddy Simulation (LES) in dealing with entropy generation analysis. They applied an approach based on a transport equation of the Filtered Density Function (FDF). The use of this method leads to a closed form of the chemical source term [23,24]. The classical filtered balance equations of mass, momentum, energy and species mass fractions are solved together with the transport equation of the filtered density function of entropy, also called entropy FDF approach (En-FDF). Such an En-FDF approach includes the statistical information regarding the scalar, velocity, turbulent frequency and entropy fields in an inclusive manner, which facilitates the formulation of the subgrid scale (sgs) closures for all non-closed terms in the filtered transport equations. The non-resolved entropy generation source terms were properly predicted in simple turbulent reacting flows, including Sandia flame D. Nevertheless, this method is expensive in terms of computational cost and not applicable, in this form, in commercial CFD codes. Recently, the authors of the present paper suggested new techniques to evaluate the entropy generation sources in addition to the classical-thermodynamics-based one: the turbulence-based approach and the look-Up-table-based approach [25]. Resting upon the investigation of Ries et al. [26], in the turbulence-based approach, the resolved turbulent quantities serve to quantify the local entropy production rates in a post-processing phase of LES. The entropy generation rates at subgrid scale are then modeled with no need to solve additional transport equations. Unlike the thermodynamic-based approach, this technique is not costly and appears applicable as an easy postprocessing tool with existing eddy-viscosity-based models. The second new presented method which is the look-up-table-based approach [25] is less costly in terms of computation. It consists of calculating and storing the entropy generation source terms as the other thermochemical properties required for simulation in the look-up table in postprocessing phase while building the 2D-FGM manifold.

In the adiabatic combustion system, the exergy losses also consider the exergy of the exhaust gases in addition to the exergy destroyed inside the combustion chamber. As stated in [19,20], the exergy content of exhaust gases accounts for a significant fraction of input exergy. A few literature studies dealing with the exergy of exhaust gases can be found [19,20,27]. Only recently has the relation between exergy and environmental impact been investigated [28,29,30,31,32,33]. The improvement in the exergy efficiency of combustion systems can help not only to improve sustainability but also the decrease in the negative environmental impact [34]. For these reasons, a deeper understanding of the exergy of exhaust gases is needed.

In the present study, the exergy losses of the Sandia flames E and F, which were considered as an adiabatic combustion system, are addressed. Previous studies treated these flames using the transported FDF approach based on the Lagrangian procedure [23,24] or on the Eulerian stochastic field in [35,36,37]. To date, exergy loss evaluation based on a transported FDF approach relying on an Eulerian stochastic field has not been made available in the literature.

The present paper aimed therefore: (a) to extend the exergy destruction analysis by including the exhaust gas exergy while using the Flamelet Generated Manifold tabulated chemistry method combined with the hybrid filtered Eulerian stochastic field (ESF) approach; (b) to propose a novel method for calculating the exergy content of exhaust gases; and (c) to point out a link between the exhaust gas exergy and the combustion emissions.

The present work is structured as follows: In Section 2, the models describing the turbulent reacting flow using the tabulated chemistry within the LES framework as well as the exergy analysis are presented. In Section 3, the two investigated flame cases are first introduced. Then, the numerical results are validated and discussed. A detailed exergy analysis in both cases is afterwards provided. Finally, Section 4 is dedicated to conclusions.

## 2. Numerical Modeling

In this section, the governing equations describing the reacting flows using the LES hybrid ESF/FGM approach and the exergy analysis through different methods for the entropy generation rates in single-phase turbulent reacting flows are introduced.

### 2.1. Eulerian Stochastic Fields Method

The LES hybrid ESF/FGM model is based on coupling the transported joint scalar filtered density function (T-FDF) following the ESF approach with the Flamelet Generated Manifold-based combustion model.

#### 2.1.1. Manifold Generation

The flamelet generated manifold (FGM), considered one of the most efficient reduction techniques, describes the detailed chemistry by only a few controlling variables. In FGM, the choice of representative flamelets and control variables depends on the combustion system to which it is being applied. In the present work, which deals with diffusion combustion systems, the mixture fraction, Z, as defined by Bilger et al. [38], and the progress variable, *Yc,* were chosen as the controlling variables. The progress variable *Yc* is given by [39]:

(1) $$Y_{c}=\frac{Y_{CO_{2}}}{M_{CO_{2}}}+\frac{Y_{CO}}{M_{CO}}+\frac{Y_{H_{2}O}}{M_{H_{2}O}}$$

where *Y* and *M* are the mass fraction and the molar mass, respectively. A set of one-dimensional diffusion flamelets of varying strain rate under unity Lewis number assumption are solved with a counter-flow flame solver from Cantera code [40]. The GRI-Mech 3.0 mechanism [23] was adopted to solve the chemical kinetics. A two-dimensional manifold was afterwards constructed by collecting the obtained flamelets. It is also known as the look-up table in which all the different thermochemical data required for the simulation are saved.

#### 2.1.2. Coupling FGM to LES

As stated in the previous section, the flamelet generated manifold is stored as a database controlled by the mixture fraction, Z, and the progress variable, *Yc*. Therefore, the reacting flow is described by the classical transport equations for mass density, momentum and by additional transport equations for all the control variables of the manifold. The set of filtered equations to be solved within the LES framework are given as:

(2) $$\frac{\partial \bar{\rho }}{\partial t}+\frac{\partial \bar{\rho }{\tilde{u}}_{i}}{\partial x_{i}}=0$$

(3) $$\frac{\partial \bar{\rho }{\tilde{u}}_{i}}{\partial t}+\frac{\partial \bar{\rho }{\tilde{u}}_{i}{\tilde{u}}_{j}}{\partial x_{j}}=-\frac{\partial \bar{p}}{\partial x_{i}}+\frac{\partial }{\partial x_{j}}\left[\bar{\mu }\left(\frac{\partial {\tilde{u}}_{i}}{\partial x_{j}}+\frac{\partial {\tilde{u}}_{j}}{\partial x_{i}}-\frac{2}{3}\frac{\partial {\tilde{u}}_{k}}{\partial x_{k}}\delta_{ij}\right)\right]\_\frac{\partial }{\partial x_{j}}\left(\bar{\rho }\tau_{ij}^{sgs}\right)$$

(4) $$\frac{\partial \bar{\rho }\tilde{Z}}{\partial t}+\frac{\partial \bar{\rho }{\tilde{u}}_{j}\tilde{Z}}{\partial x_{j}}=\frac{\partial }{\partial x_{j}}\left[\left(\frac{\bar{\mu }}{Sc}+\frac{\mu_{sgs}}{Sc_{sgs}}\right)\frac{\partial \tilde{Z}}{\partial x_{j}}\right]$$

(5) $$\frac{\partial \bar{\rho }{\tilde{Y}}_{c}}{\partial t}+\frac{\partial \bar{\rho }{\tilde{u}}_{j}{\tilde{Y}}_{c}}{\partial x_{j}}=\frac{\partial }{\partial x_{j}}\left[\left(\frac{\bar{\mu }}{Sc}+\frac{\mu_{sgs}}{Sc_{sgs}}\right)\frac{\partial {\tilde{Y}}_{c}}{\partial x_{j}}\right]+{\bar{\dot{\omega }}}_{Yc}$$

The quantities ρ, *u*, *p* and *µ*, in Equations (2)–(5), are the density, the velocity, the pressure, the dynamic molecular viscosity of the fluid, respectively. The terms *δij* and $\tau_{ij}^{sgs}$ stand for the Kronecker delta and the sub-grid scale stress tensor. Note that $\bar{\left(.\right)}$, $\tilde{\left(.\right)}$ and (.)*sgs* denote the filtered, Favre filtered and sub-grid scale quantities, respectively. In this paper, the modeling of the sub-grid scale to describe the effect of the unresolved small eddies is performed using the Sigma eddy-viscosity model [41]. To avoid repetition, the details of this model are not provided herein; please instead refer to our previous paper [25].

As the reaction source term *ω_Yc_*, in Equation (5) is highly nonlinear and remains normally unclosed in an LES context, it is difficult to accurately and correctly represent the thermochemical state only with the LES-filtered values of the controlling variables. Thus, the turbulence–chemistry interaction at the sub-grid scale level must be accounted for. In order to accomplish this task and provide the chemical source term in a closed form, the Eulerian stochastic fields (ESF) method was adopted.

#### 2.1.3. The Eulerian Stochastic Field (ESF) Method

The general concept of the ESF approach was introduced by Valińo [42] (see also Dopazo [43]). It was updated and used in [35,36,37] to solve the transport equation for the filtered joint probability density function, also denoted FDF for controlling variables. The conservation equation for the joint probability density function $\tilde{P}$(*Ψ*) in the LES context reads as [21,35,36,37,39,44,45,46]:

(6) $$\begin{array}{l} \underset{I}{\underbrace{\frac{\partial \bar{\rho }\tilde{P}\left(\psi \right)}{\partial t}}}+\underset{II}{\underbrace{\frac{\partial \bar{\rho }{\tilde{u}}_{j}\tilde{P}\left(\psi \right)}{\partial x_{j}}}}-\underset{III}{\underbrace{\sum_{\alpha =1}^{N_{\alpha }}\frac{\partial \left(\bar{\rho }{\dot{\omega }}_{\alpha }\tilde{P}\left(\psi \right)\right)}{\partial \psi_{\alpha }}}}=\\ -\underset{IV}{\underbrace{\frac{\partial }{\partial x_{i}}\left[\left(\left({\tilde{\rho u}}_{i}-\bar{\rho }{\tilde{u}}_{i}|\phi =\psi \right)\right)\tilde{P}\left(\psi \right)\right]}}-\underset{V}{\underbrace{\sum_{\alpha =1}^{N_{\alpha }}\sum_{\beta =1}^{N_{\beta }}\frac{\partial^{2}}{\partial \psi_{\alpha }\partial \psi_{\beta }}\left[\bar{\left(\frac{\mu }{Sc}\frac{\partial \phi_{\alpha }}{\partial x_{i}}\frac{\partial \phi_{\beta }}{\partial x_{i}}|\phi =\psi \right)}\tilde{P}\left(\psi \right)\right]}} \end{array}$$

The FDF transport equation (Equation (6)) involves the variation in the physical space (term I), the convective transport on the resolved scales (term II) and the chemical reaction source which appears in closed form in the phase space (term III). In the RHS of Equation (6), the term IV stands for the turbulent transport and lastly, the micro-mixing (molecular diffusion) is accounted for by term V. These last two terms which appear unclosed need to be modeled. Within this work, the transport of the sub-grid FDF is modeled as is the momentum transport equation, which considers a gradient assumption along with an eddy-diffusivity with a turbulent Schmidt number *Sc_sgs_* = 0.7 [44]. The linear mean square estimation closure (LMSE) [43,47,48], also reported under the interaction by exchange with the mean model (IEM) [49], was adopted to provide a closure for the molecular diffusion or the micro-mixing term.

For the purpose of solving the joint probability density function at the sub-grid scale of the controlling variables, the FDF is constructed from a number of Eulerian stochastic fields, Ns. The composition of each controlling variable $\alpha =\left\{Y_{C},Z\right\}$ is included in every stochastic field $\xi_{\alpha }^{n}\left(x_{i},t\right)$. These fields can be expressed as follows (e.g., [15,16,17,39,44]):

(7) $$\begin{array}{l} d\left(\bar{\rho }\xi_{\alpha }^{n}\right)=-\frac{\partial }{\partial x_{j}}\left(\bar{\rho }\xi_{\alpha }^{n}u_{j}\right)dt+\frac{\partial }{\partial x_{i}}\left[\left(\frac{\bar{\mu }}{Sc}+\frac{\mu_{sgs}}{Sc_{sgs}}\right)\frac{\partial \xi_{\alpha }^{n}}{\partial x_{i}}\right]dt+\bar{\rho }{\dot{\omega }}_{\alpha }^{n}dt\\ -\frac{\bar{\rho }}{2\tau_{{}_{t}}}\left(\xi_{\alpha }^{n}-{\tilde{\phi }}_{\alpha }\right)dt+\bar{\rho }\sqrt{\frac{2}{\bar{\rho }}\frac{\mu_{sgs}}{Sc_{sgs}}}\frac{\partial \xi_{\alpha }^{n}}{\partial x_{i}}dW_{j,\alpha }^{n}\;\;\;\alpha =\left\{Z,\;PV\right\};\;n=\left(1,\;2,\;\ldots ,\;Ns\right)\; \end{array}$$

where *τ_t_* is the micro mixing time scale defined as (e.g., [35,36,37,44,46,47]):

(8) $$\tau_{t}=\frac{1}{\Omega }=C_{\Omega }\frac{\upsilon +\upsilon_{sgs}}{\Delta^{2}}$$

In Equation (8), ∆ and *ν* stand for the grid filter width and kinematic viscosity, respectively. The micro-mixing constant is, following Avdic et al. [46], defined as *C*_Ω_ = 2.

The last term in the RHS of Equation (7) represents the stochastic Wiener term to describe the effect of the turbulent diffusion at the sub-grid level. The quantity $dW_{\alpha }^{n}$, the vector Wiener process, defined as $dW_{\alpha }^{n}=\eta_{\alpha }^{n}\sqrt{\Delta t}$ spatially uniform and different for each stochastic field is proposed. The Wiener process is known as a random walk, normally distributed with zero mean and variance of the incremental time ∆t for Ns stochastic fields. However, for a low number of stochastic fields, sampling the components of the vector increments $\eta_{\alpha }^{n}$ of a normal distribution will hardly meet these constraints. Therefore, a weak first-order approximation is considered in which the increments are sampled from a dichotomic distribution {−1, 1} [50]. In fact, this approximation is not enough to accurately provide the correct mean and variance. To avoid this problem, a complementary increment $\eta_{\alpha }^{i+Ns/2}=-\eta_{\alpha }^{n}$ for the first half of the stochastic increments is used before randomly shuffling the set to prevent any correlation between$\;\eta_{\alpha }^{n}$ and$\;\eta_{\alpha }^{i+Ns/2}$ [51]. Finally, the filtered mean of the variable ϕ*_α_* is obtained through the first moment and its sub-grid variance by the second moment as:

(9) $${\tilde{\phi }}_{\alpha }=\frac{1}{N_{s}}\sum_{n=1}^{N_{s}}\xi_{\alpha }^{n};\;\phi_{\alpha ,sgs}=\frac{1}{N_{s}}\sum_{n=1}^{N_{s}}{\left(\xi_{\alpha }^{n}-{\tilde{\phi }}_{\alpha }\right)}^{2}$$

#### 2.1.4. Numerical Solution Procedure

A new solver based on the ESF-FGM approach, implemented in OpenFOAM code, was used to carry out all the simulations. However, the numerical instabilities induced by the stochastic fluctuations of the density and its derivative and which occurred during the solution, especially for a low number of stochastic fields, were very challenging for this solver [52]. Within this work, the so-called auxiliary moments of the progress variable, Yc*, and the mixture fraction, Z*, were introduced to reduce these stochastic fluctuations [35]:

(10) $$\frac{\partial \bar{\rho }{\tilde{Z}}^{*}}{\partial t}+\frac{\partial \bar{\rho }{\tilde{u}}_{j}{\tilde{Z}}^{*}}{\partial x_{j}}=\frac{\partial }{\partial x_{j}}\left[\left(\frac{\bar{\mu }}{Sc}+\frac{\mu_{sgs}}{Sc_{sgs}}\right)\frac{\partial {\tilde{Z}}^{*}}{\partial x_{j}}\right]$$

(11) $$\frac{\partial \bar{\rho }{\tilde{Y}}_{c}^{*}}{\partial t}+\frac{\partial \bar{\rho }{\tilde{u}}_{j}{\tilde{Y}}_{c}^{*}}{\partial x_{j}}=\frac{\partial }{\partial x_{j}}\left[\left(\frac{\bar{\mu }}{Sc}+\frac{\mu_{sgs}}{Sc_{sgs}}\right)\frac{\partial {\tilde{Y}}_{c}^{*}}{\partial x_{j}}\right]+\frac{1}{N_{s}}\sum_{n=1}^{N_{s}}{\dot{\omega }}_{Yc}^{n}$$

The solution of Equations (10) and (11) for the auxiliary control variables serves to compute filtered density ρ* and viscosity *µ*^∗^, which are continuously utilized in all equations to be solved. Following the solution steps explained in [25,44] and preserving the main numerical setup in terms of the different numerical scheme, the momentum predictor, pressure solver and stochastic contribution were not considered in the first phase of solution procedure while the stochastic fields were computed. In the second phase of calculation, when all fields reached convergence, the respective stochastic terms were included [53]. Simulations were performed using adjustable time step ∆*t* around 10^−7^ to maintain the CFL-number below unity. As stated in previous works [35,36,37,44], eight stochastic fields are found sufficient to reach the convergence for Sandia flames.

### 2.2. The Exergy Losses of Adiabatic Turbulent Flame

The exergy analysis approaches are promising techniques for increasing fuel conversion efficiency as it is good at dealing with the irreversibilities associated with the real energy conversion processes. The quantification of the energy degradation, also called the loss of exergy, stems from irreversibilities in the combustion system and begins with the definition of the exergy balance for the control volume as follows [19,54]:

(12) $$\sum {\dot{m}}_{in}\dot{E}x_{in}=\dot{E}x_{work}+\dot{E}x_{dest}+\dot{E}x_{heat}+\dot{E}x_{exhaust}$$

where ${\dot{Ex}}_{in}$ is the exergy input rate consisting of fuel exergy and combustion air exergy, ${\dot{Ex}}_{work}$ expresses the useful net work, ${\dot{Ex}}_{dest}$ represents the exergy destruction rate during the combustion, ${\dot{Ex}}_{heat}$ is the exergy transfer rate of the heat transferred to the environment and ${\dot{Ex}}_{exhaust}$ accounts for the exergy output rate at the exhaust. The last three terms in the RHS of Equation (12) are the sources of exergy destruction in the combustion system. Within this work, we are dealing with adiabatic diffusion turbulent flames with no differential diffusion considered. Thus, the only exergy destruction during combustion ${\dot{Ex}}_{dest}$ and the exergy content of the exhaust ${\dot{Ex}}_{exhaust}$ are considered.

#### 2.2.1. Exergy Losses during Combustion

Exergy destruction during combustion is evaluated through entropy generation as follows:

(13) $$\dot{E}x_{dest}=T_{0}\Pi_{g}$$

where *T*_0_ is the ambient temperature and Π*_g_* is the total rate of the entropy generation rate induced by the irreversibilities of the processes involved in the combustion system. The entropy production rate is derived from the filtered transport equation of entropy within the combustion system. Considering the gradient assumption for the entropy diffusion term, according to [22], it yields:

(14) $$\begin{array}{l} \frac{\partial \bar{\rho }\tilde{s}}{\partial t}+\frac{\partial }{\partial x_{i}}\left(\bar{\rho }{\tilde{u}}_{i}\tilde{s}\right)=\frac{\partial }{\partial x_{i}}\left(\bar{\rho }D_{m}\frac{\partial \tilde{s}}{\partial x_{i}}\right)-\frac{\partial }{\partial x_{i}}\left(\bar{\rho }\tau \left(u_{i},s\right)\right)\\ +\underset{\Pi_{v}}{\underbrace{\bar{\frac{1}{T}\tau_{ij}\frac{\partial u_{i}}{\partial x_{j}}}}}+\underset{\Pi_{q}}{\underbrace{\bar{\frac{\lambda }{T^{2}}\frac{\partial T}{\partial x_{i}}\frac{\partial T}{\partial x_{i}}}}}+\underset{\Pi_{d}}{\underbrace{\bar{\frac{\lambda }{c_{p}}\sum_{k=1}^{N}\frac{R_{k}}{Y_{k}}\frac{\partial Y_{k}}{\partial x_{i}}\frac{\partial Y_{k}}{\partial x_{i}}}}}-\underset{\Pi_{ch}}{\underbrace{\bar{\frac{1}{T}\sum_{k=1}^{N}\mu_{k}{\dot{\omega }}_{k}}}} \end{array}$$

where *D_m_* denotes the diffusion coefficient and *τ* (*a*, *b*) are the second-order SGS moments given by:

(15) $$\tau \left(a,b\right)=\tilde{ab}-\tilde{a}\tilde{b}$$

In Equation (14), the first two terms on the LHS accounted for the accumulation and convection process, respectively. On the RHS, the molecular and turbulent diffusion of entropy is described by the first two terms and the total entropy generation within the flame is the sum of the last four terms. The involved processes engendering the entropy production are mainly: viscous dissipation (II_*v*_), heat transfer (II_*q*_), mass/diffusion of species (II_*d*_) and chemical reaction (II_*ch*_). Additional modeling is required to provide closure for these terms. In Equation (14), *λ*, *C_p_*, *R_k_* and *μ_k_* are the thermal conductivity, specific heat capacity, gas constant of species and specific chemical potential of species, respectively. It is worth mentioning that, in Equation (14), the cross-terms in the gradient-based contributions of the entropy production are not considered.

In our previous work [25], three different approaches to model these entropy generation source terms were presented and validated: the thermodynamics-based approach, turbulence-based approach and the look-up table-based approach. Good agreement was observed between these different methods, and thus we chose the less expensive in terms of computational cost for this work, namely the look-up table-based approach [25]. Therefore, while building the 2D manifold, the entropy generation source terms were computed for each 1d flame. The obtained flamelets with the computed entropy production source terms were then saved in the look-up table to be used during calculation. The 2D-FGM is controlled by two variables (the mixture fraction and the progress variable)—which allows the application of the partial differentiation rule to obtain the derivatives including in the different entropy production source terms (not filtered) as follows:

(16) $$\Pi_{q}=\frac{\lambda }{T^{2}}\frac{\partial T}{\partial x_{i}}\frac{\partial T}{\partial x_{i}}=\frac{\lambda }{T^{2}}\left[{\left(\frac{\partial T}{\partial Y_{c}}\frac{\partial Y_{c}}{\partial x_{i}}\right)}^{2}+2\frac{\partial T}{\partial Y_{c}}\frac{\partial T}{\partial Z}\frac{\partial Y_{c}}{\partial x_{i}}\frac{\partial Z}{\partial x_{i}}+{\left(\frac{\partial T}{\partial Z}\frac{\partial Z}{\partial x_{i}}\right)}^{2}\right]$$

(17) $$\Pi_{d}=\frac{\lambda }{c_{p}}\sum_{k=1}^{N}\frac{R_{k}}{Y_{k}}\frac{\partial Y_{k}}{\partial x_{i}}\frac{\partial Y_{k}}{\partial x_{i}}=\frac{\lambda }{c_{p}}\left[\sum_{k=1}^{N}\frac{R_{k}}{Y_{k}}{\left(\frac{\partial Y_{k}}{\partial Y_{c}}\frac{\partial Y_{c}}{\partial x_{i}}\right)}^{2}+\sum_{k=1}^{N}2\frac{R_{k}}{Y_{k}}\frac{\partial Y_{k}}{\partial Y_{c}}\frac{\partial Y_{k}}{\partial Z}\frac{\partial Y_{c}}{\partial x_{i}}\frac{\partial Z}{\partial x_{i}}+\sum_{k=1}^{N}\frac{R_{k}}{Y_{k}}{\left(\frac{\partial Y_{k}}{\partial Z}\frac{\partial Z}{\partial x_{i}}\right)}^{2}\right]$$

(18) $$\Pi_{ch}=-\frac{1}{T}\sum_{k=1}^{N}\mu_{k}{\dot{\omega }}_{k}$$

Finally, the entropy generated from the viscous dissipation is treated similarly as in the turbulence-based method [25]:

(19) $${\bar{\Pi }}_{v}=\frac{\bar{\rho }\bar{\nu }}{\bar{T}}\left(\frac{\partial {\bar{u}}_{i}}{\partial x_{j}}+\frac{\partial {\bar{u}}_{j}}{\partial x_{i}}\right)\frac{\partial {\bar{u}}_{i}}{\partial x_{j}}+\frac{\bar{\rho }}{\bar{T}}{є}_{k,sgs}\;;\;{є}_{k,sgs}=\frac{1}{\Delta^{4}C_{s}^{4}}\upsilon_{sgs}{}^{3}$$

where *Cs* = 0.17 is the Smagorinsky constant.

#### 2.2.2. Exergy Losses at the Exhaust Gas

The exergy associated with the exhaust gases generated after the combustion flowing at mass flow ${\dot{m}}_{exhaust}$ through the exhaust manifold includes two different components, namely the physical exhaust exergy ${\dot{Ex}}_{exhaust,ph}$ and the chemical exhaust exergy ${\dot{Ex}}_{exhaust,ch}$ [19,54]:

(20) $$\dot{E}x_{exhaust}=\dot{E}x_{exhaust,ph}+\dot{E}x_{exhaust,ch}$$

The physical exhaust exergy ${\dot{Ex}}_{exhaust,ph}$, also called thermo-mechanical exergy, which is associated with the exhaust gases pressure, $P_{exhaust}$, and temperature, $T_{exhaust}$—which is much higher compared to that of atmospheric temperature ($T_{0}$) [55]—is defined as:

(21) $$\dot{E}x_{exhaust,ph}={\dot{Q}}_{exhaust}+{\dot{m}}_{exhaust}T_{0}\left[C_{p,exhaust}\ln\left(\frac{T_{0}}{T_{exhaust}}\right)-R_{exhaust}\ln\left(\frac{P_{0}}{P_{exhaust}}\right)\right]$$

where ${\dot{Q}}_{exhaust}$ is the heat energy taken by exhaust gases and expressed by means of the mass flow rate, specific heat and temperature of the exhaust gases as follows:

(22) $${\dot{Q}}_{exhaust}={\dot{m}}_{exhaust}C_{p,exhaust}\left(T_{exhaust}-T_{0}\right)$$

The second term of the exhaust exergy, which is the chemical exhaust exergy, ${\dot{Ex}}_{exhaust,ch}$, is evaluated as [19,54,56,57,58]:

(23) $$\dot{E}x_{exhaust,ch}={\dot{m}}_{exhaust}RT_{0}\sum \ln\frac{Y_{i}}{Y_{ir}}$$

where *R* and $T_{0}\;$are the universal gas constant and the temperature of the reference ambient state, respectively, whereas $Y_{i}$ is the molar fraction of the ith species in the exhaust gas and $Y_{ir}\;$is the molar fraction of the same ith species in the reference environment. In this work, these reference values of molar fraction, $Y_{ir}$, are defined in [59].

Both parts of the exergy content of exhaust gases were calculated using the look-up table approach as well as the related entropy source term. Using this approach, the computation of the exergy of the exhaust gas will not be costly in terms of computational cost since in this case, there will be no need for the calculation of the mass fractions of all species of exhaust gases during the simulation. The exergy of exhaust gases will be tabulated as well as all the other thermophysical data required for flame computation.

## 3. Results and Discussion

### 3.1. Experimental and Numerical Setup

In this work, Sandia flames E and F were investigated using the LES hybrid ESF/FGM method with eight stochastic fields to assess the thermodynamic efficiency through the estimation of exergy losses. The studied flames share the same burner configuration but different boundary conditions, as recently outlined by the authors of the present paper in [25]. They are composed of three inlets streams, namely the fuel and the pilot inlets as well as the co-flow, as described in [25]. The fuel inlet, a mixture of 25% methane and 75% air by volume, is entering through a jet of the diameter, d of 7.2 mm at 290 K with an average velocity of 74.4 and 99.2 m/s corresponding to flames E and F, respectively. Through the pilot, in the second inlet of an outer diameter of 18.2 mm, flows the product of the lean pre-combustion of methane with air at a temperature of 1880 K and with a mean flow velocity of 17.1 and 22.8 m/s for flames E and F, respectively. The pilot is then surrounded by a co-flow at approximately 0.9 m/s. A detailed description of the burner geometry and the boundary conditions can be found in [25,37]. The data from the measurements of various scalars and velocities are provided in [60].

A numerical grid of size of 2.9 million cells was used to carry out the calculations. Note that a 3D structured hexahedral created mesh was well refined near the walls and in the fuel jet zone with a smallest cell of 10^−11^ m^−3^ in order to enhance the accuracy in computing the flow and scalar gradients, and consequently, the entropy generation rates.

The turbulent inlet at the burner inlets was provided by the use of the synthetic turbulence and mapping techniques. This consists of mapping the fuel inlet velocity at the cross plane 5.5d (d is the diameter of the fuel inlet) downstream onto the inlet fuel plane. However, the synthetic turbulence was applied to the pilot inlet. Lastly, the atmospheric pressure was considered at the inlet and outlet boundary conditions.

### 3.2. Validation

**Figure 1:**
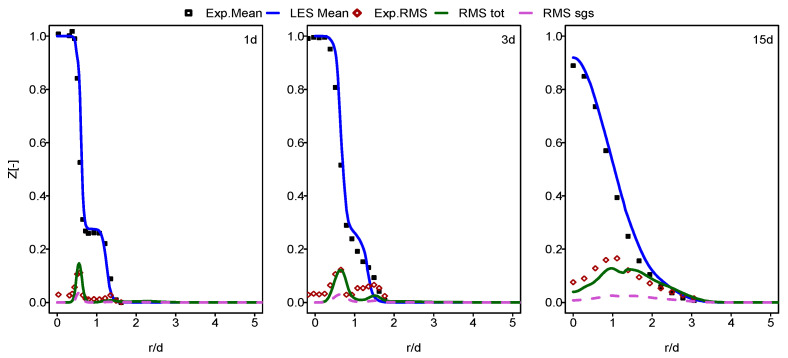
Mean and RMS mixture fraction at different axial positions: case of flame E. Dashed line: unresolved contribution from LES/ESF to RMS.

**Figure 2:**
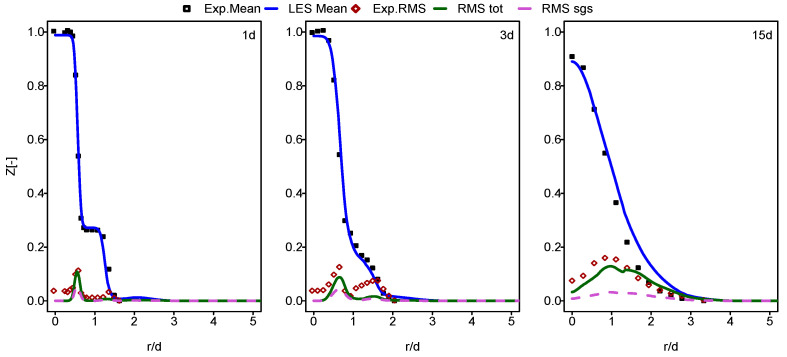
Mean and RMS mixture fraction at different axial positions: case of flame F.

**Figure 3:**
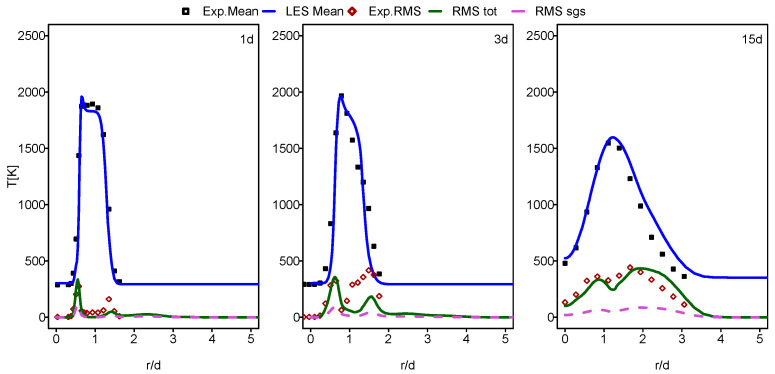
Mean and RMS temperature at different axial positions: case of flame E.

**Figure 4:**
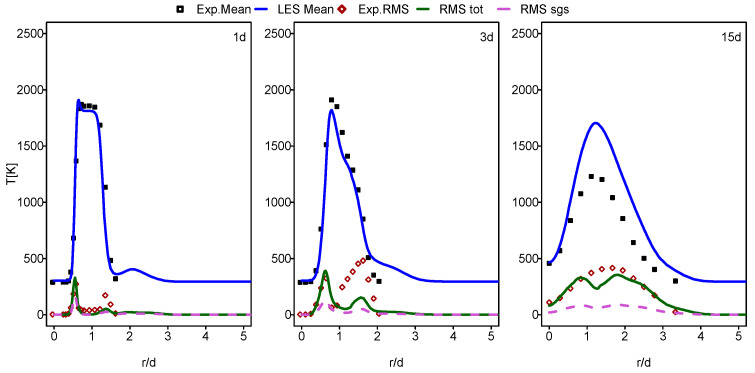
Mean and RMS temperature at different axial positions: case of flame F.

In this part of the paper, the simulation results for Flames E and F are presented and compared with the experimental data provided in [60]. In Figure 1, Figure 2, Figure 3 and Figure 4, the mean and RMS radial profiles of the mixture fraction and the temperature at the three axial positions, 1d, 3d and 15d for Sandia E and F, are displayed. Additionally, the time-averaged subgrid contributions provided by the ESF calculation are also presented (displayed as dashed lines). The mixture fraction results depicted in Figure 1 and Figure 2 for Sandia E and F, respectively, show better agreement for flame E compared to flame F, especially far away from the burner inlet. This is clearly visible in the temperature results shown in Figure 3 and Figure 4. It seems that eight stochastic fields were enough to provide the higher accuracy of the simulation of the flame E [25,36,37] and not enough for the simulation of flame F [36,37]. At axial position 15d, the mean temperatures for Flame F are over predicted by a quite larger margin compared to Flame E. However, these discrepancies are not intense for a mean mixture fraction. For both cases, some underestimations for RMS values, especially at the position 3d, were detected.

### 3.3. Exergy Losses Analysis

This section is dedicated to the analysis of the exergy losses for Sandia E and F considered as adiabatic turbulent flames. As stated in this section, the main exergy losses within an adiabatic combustion system are the exergy destroyed inside the combustion chamber during combustion and the exergy content of exhaust gases. The exergy losses inside the combustion chamber were computed by quantifying the entropy generation enhanced by the various sources of irreversibility intrinsically associated with the different processes involved in these two turbulent flames. To obtain these different entropy generation source terms, the look-up table-based approach was used as detailed in Section 2.2.1.

#### 3.3.1. Entropy Generation during Combustion

**Figure 5:**
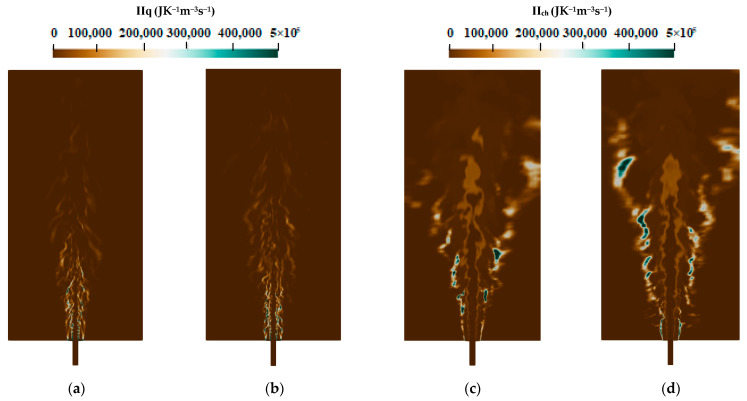
Instantaneous entropy generation due to heat transfer: (**a**) flame E; (**b**) flame F and chemical reaction; (**c**) flame E; and (**d**) flame F.

**Figure 6:**
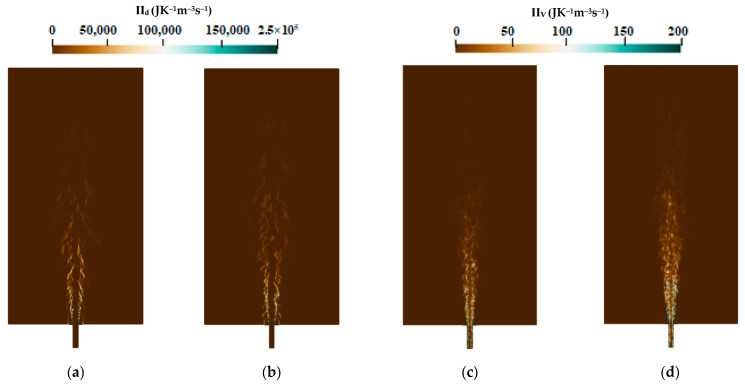
Instantaneous entropy generation due to mass diffusion (**a**) flame E; (**b**) flame F and viscous dissipation; (**c**) flame E; and (**d**) flame F.

The instantaneous contour plots of the different entropy generation source terms for flames E and F are presented in Figure 5 and Figure 6. For both cases, the heat transfer and the chemical reaction shown in Figure 5a–d are the main responsible factors for the entropy production, i.e., the exergy destruction. Flames E and F show the same behavior as in entropy generation distribution: higher entropy values are generated due to the heat transfer, which are mainly located in the jet zone and the nozzle exit; however, the entropy produced by the chemical reaction is found in the flame zone and far away downstream the burner. The contribution of mass diffusion and the viscous dissipation presented as depicted in Figure 6a–d seems to be lower compared to those of the other processes. Nevertheless, the mass diffusion contributes more than the chemical reaction to the entropy production near the nozzle exit (at 1d position), which is reinforced with Flame F compared to the Flame E since the jet mass flow is increased which increases entropy generation due to viscous dissipation.

**Figure 7:**
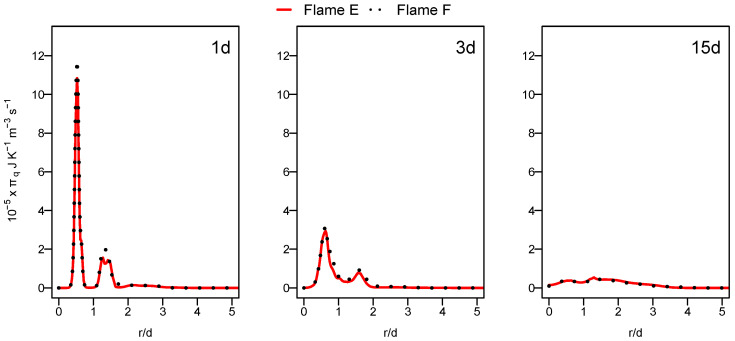
Radial profile of the volumetric entropy from heat transfer at various axial positions for flame E (red) and flame F (black dashed).

Figure 7 displays the radial profiles of the heat transfer entropy generation source term for flames E and F. Both flames show comparable value with a slight increase at position 1d for flame F. The over prediction of the temperature of flame F, which is far away from the burner exit shown in the previous section in Figure 4, caused this comparable behavior of entropy generation by heat transfer for both flames. The increase in the Reynolds number with flame F should normally lead to the increase in the presence of ex-tinction pockets compared to flame E which will rise the temperature gradient, resulting in an increase in entropy generation. This is not visible here because the application of the ESF method with eight stochastic field was not sufficient for flame F.

**Figure 8:**
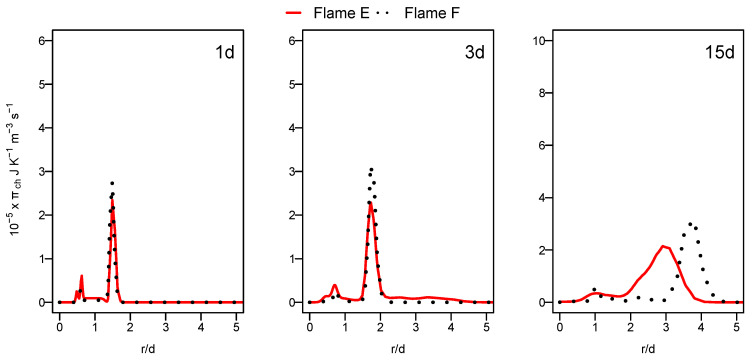
Radial profile of the chemical reaction entropy generation source term at different axial positions for flames E and F.

Unlike the heat transfer entropy source term, the chemical reaction entropy source term, as depicted in Figure 8, is higher for flame F compared to flame E, especially at a downstream location (see 15d position). The increase in the mixing rate caused by the increased jet and pilot and the mass flow rate augments the species concentration gradients, which leads to the increase in entropy production in the case of flame F compared to flame E. In both cases, the contribution of the chemical reaction remains lower than that of the heat transfer term.

**Figure 9:**
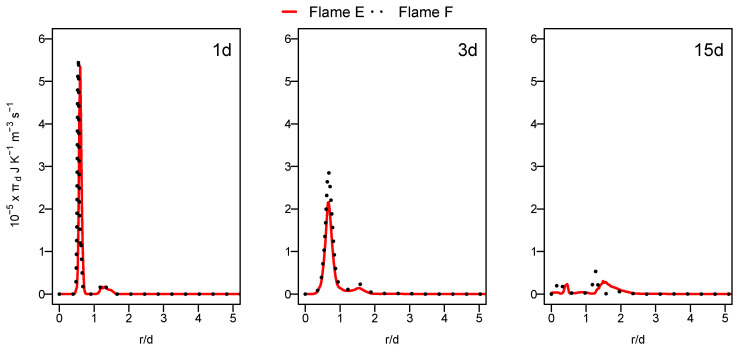
Radial profile of the mass diffusion entropy generation source term at various axial positions for flames E and F.

The entropy production from mass diffusion as presented in Figure 9 shows an increase for flame F compared to flame E downstream the burner. The increase in the inlet mass flow from flame E to F leads to the enhancement of the species diffusion which consequently rises the entropy generated from the mass diffusion. The difference in entropy produced by mass diffusion between the two flames in terms of values may be deeper downstream the burner if the flow and scaler fields are more accurately predicted for the flame F. At the nozzle exit and near the inlet, the mass diffusion has higher contribution in entropy generation than the chemical reaction but still lower than the heat transfer entropy source term.

**Figure 10:**
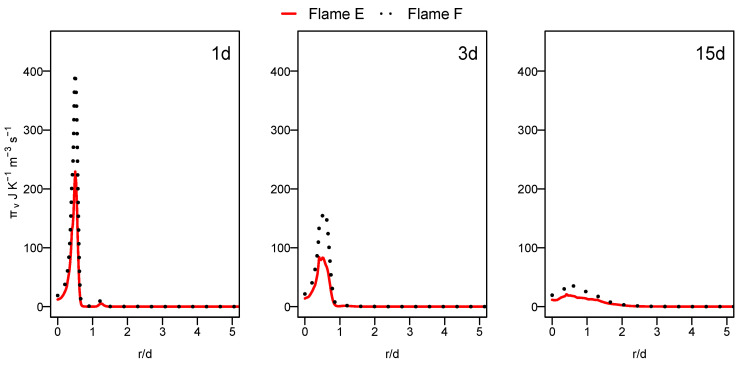
Radial profile of the viscous dissipation entropy generation source term at different axial positions for flames E and F.

Increasing the jet velocity increases the viscous dissipation contribution of the flame F compared to flame E, as shown by Figure 10. However, even with these highest Reynolds values, the entropy generation due to the viscous dissipation is always insignificant compared to other processes.

To summarize, from Figure 7, Figure 8, Figure 9 and Figure 10, it is clear that the major contribution in entropy generation is attributed to heat transfer. However, a competition was found between the chemical reaction and mass diffusion, especially by increasing the jet velocity with flame F.

#### 3.3.2. Exhaust Gases Exergy

In this section, the exergy content of the exhaust gases will be investigated. The focus will be on the chemical exergy content of exhaust gases. As stated in Section 2.2.2, the mass fraction of species in exhaust gases is needed to compute the chemical exergy content. With the use of the look-up table approach, the chemical exergy of the exhaust gases was calculated as well as the different entropy generation source terms in the postprocessing step during the construction of the 2D-FGM manifold. In this case, the chemical exhaust exergy will be saved in the look-up table as well as the thermochemical data required for the combustion simulation. In this study, the exhaust was considered here at the outlet of the combustion chamber.

**Figure 11:**
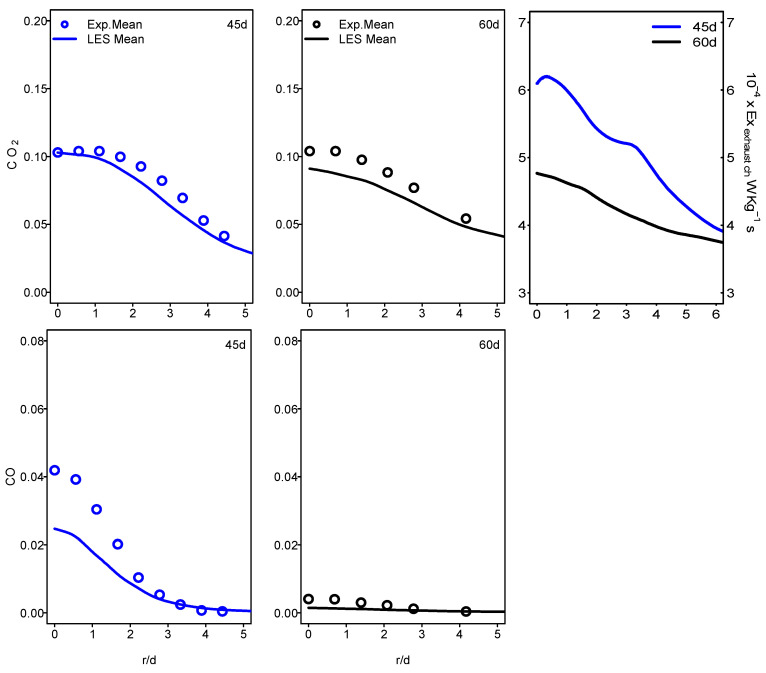
Radial profiles of mean CO_2_, CO mass fraction and chemical exhaust gases exergy at various axial locations for Sandia flame E.

**Figure 12:**
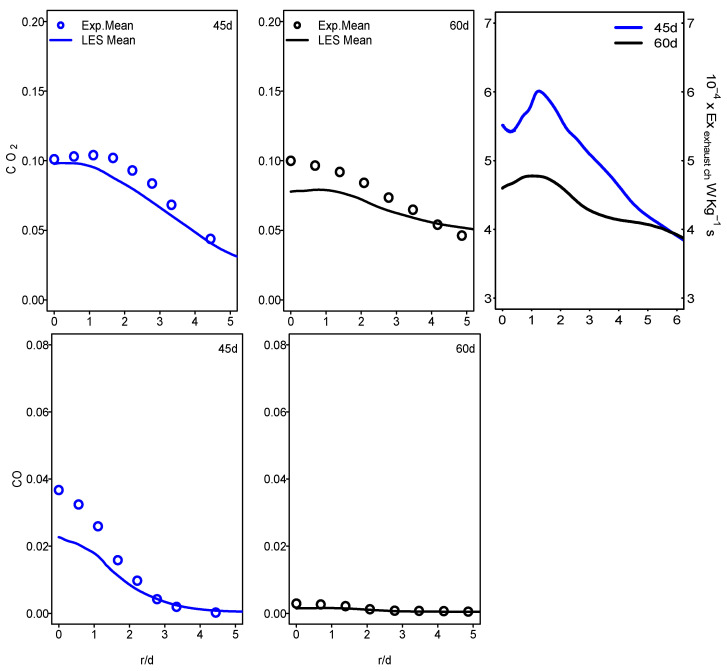
Radial profiles of mean CO_2_, CO mass fraction and chemical exhaust gases exergy at various axial locations for Sandia flame F.

**Figure 13:**
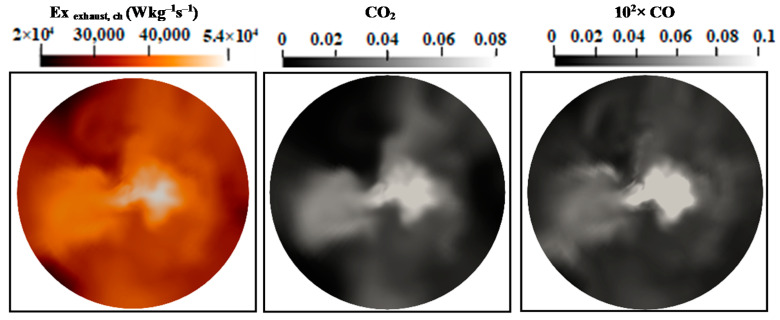
Instantaneous contour plots of the chemical exhaust gases exergy, CO_2_ and CO mass fractions Sandia flame E.

**Figure 14:**
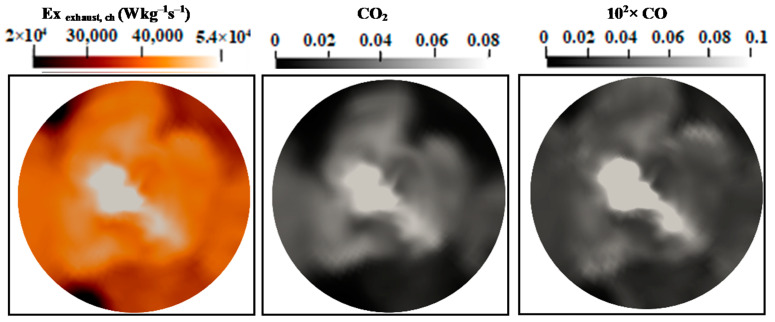
Instantaneous contour plots of the chemical exhaust gases exergy, CO_2_ and CO mass fractions Sandia flame F.

Figure 11 and Figure 12 show the mean values of the chemical exhaust at the outlet (axial position 60d) as well as upstream at the axial position 45d for flames E and F. It seems that, as one approaches the outlet, the chemical exergy of the exhaust gases decreases and higher values were detected in the main flow region. Downstream from the burner, the temperature decreases and the chemical exergy of exhaust gases also decreases, which may incite the idea of cooling the exhaust gases to reach the maximum recovery of the chemical exergy content. In addition, the behavior of the chemical exergy of the exhaust gases follows those of species’ mass fractions in the exhaust gases. Despite the under-estimation of the ESF method of the mass fraction of CO_2_ and CO which can also be found in [36,37], the evaluation of these species draws on the evolution of the chemical exergy of exhaust gases. This is also visible in Figure 13 and Figure 14, where the instantaneous contour plots of the mass fraction of CO_2_ and CO as well as the chemical exergy of exhaust gases for both flames were presented. In terms of values, the chemical exergy of the exhaust gases of flame F is higher than that of flame E, which is related to the exhaust gases species mass fractions. From these results, a strong link can be built between the combustion emissions presented by the exhaust gases and the exergy of the exhaust gases. The chemical exergy of the exhaust gases can give an idea of the combustion emissions, and as its value decreases, these emissions decrease.

## 4. Conclusions

An exergy analysis of Sandia flames E and F was investigated based on the hybrid ESF/FGM approach coupled with LES. The accuracy of this approach seems to be higher with eight stochastic fields for flame E in contrast to flame F which in reality presents more partial flame-out [36,37]. Thus, more work is required to increase the accuracy of the method to be more able to detect such an extinction and blow-off state.

The main novelties of this paper are firstly that exergy losses in an adiabatic combustion system can be easily computed at a lower computational cost using the look-up table-based approach; and secondly, the combustion emissions may be controllable with the chemical exhaust exergy without the need for computing them. Through the exergy analysis of flames E and F, the following inferences can be made:

1. The exergy destroyed inside the combustion chamber increases with the increase in the mass flow rate along with the Re-number for flame F in comparison to flame E.
  - The heat transfer and chemical reaction processes have higher contributions in entropy production compared to those of mass diffusion and viscous dissipation.
  - With the increase in the jet velocity for flame F, inducing more concentrations and temperature gradients, further increase in entropy generation was expected compared to flame E. However, the lower predictivity of the ESF in the case of flame F leads to a slight difference in entropy generation, especially for the heat transfer entropy source term.
2. The analysis of the chemical exergy content of exhaust gases decreases, going towards the combustion chamber outlet.
  - Downstream from the burner, the temperature continues to decrease. The same decrease in the chemical exergy of the exhaust gases can be related to the temperature. This leads to the fact that cooling the exhaust gases can increase the exhaust gases exergy recovery.
  - A strong link was found between the combustion emissions and the chemical exergy of the exhaust gases since its evolution follows the mass fractions of exhaust gases species.

In this paper, the LES hybrid ESF/FGM approach with eight stochastic fields seems to be not efficient for computing Sandia F despite its great accuracy compared to Sandia D and E found in our previous study [25]. Further work is required to ameliorate the predictivity of this approach in the presence of several partial flameouts to complete the study of the physical exergy of exhaust gases together with a chemical one outside the combustion chamber, far away from the outlet, and to extend the exergy losses analysis to the non-adiabatic combustion systems with more attention to the near-wall treatment with regard to the entropy generation.

## Data Availability

Not applicable.
